# Supplementary material for: Resolving the full spectrum of human genome variation using Linked-Reads
Source: Genome Res. 2019 Apr;29(4):635–45. doi: 10.1101/gr.234443.118 (PMC6442396; doi:10.1101/gr.234443.118)
Supplement: Supplemental Material [file supp_gr.234443.118_Supplemental_longranger-2.2.zip › longranger-2.2.tar.bz2!/lr2.2-public/lib/stan/cmdstan/stan/doc/api/html/expression07__grammar__def_8hpp_source.html]

Stan: src/stan/gm/grammars/expression07\_grammar\_def.hpp Source File


|  |  |
| --- | --- |
|  | Stan  2.5.0  probability, sampling & optimization |


- Main Page
- Related Pages
- Namespaces
- Classes
- Files

- File List
- File Members

All Classes Namespaces Files Functions Variables Typedefs Enumerations Enumerator Friends Macros Pages

- src
- stan
- gm
- grammars

expression07\_grammar\_def.hpp

Go to the documentation of this file.

1 #ifndef STAN\_\_GM\_\_PARSER\_\_EXPRESSION\_GRAMMAR07\_DEF\_\_HPP\_\_

2 #define STAN\_\_GM\_\_PARSER\_\_EXPRESSION\_GRAMMAR07\_DEF\_\_HPP\_\_

3

4 #include <cstddef>

5 #include <iomanip>

6 #include <iostream>

7 #include <istream>

8 #include <map>

9 #include <set>

10 #include <sstream>

11 #include <string>

12 #include <utility>

13 #include <vector>

14 #include <stdexcept>

15

16 #include <boost/spirit/include/qi.hpp>

17 // FIXME: get rid of unused include

18 #include <boost/spirit/include/phoenix\_core.hpp>

19 #include <boost/spirit/include/phoenix\_function.hpp>

20 #include <boost/spirit/include/phoenix\_fusion.hpp>

21 #include <boost/spirit/include/phoenix\_object.hpp>

22 #include <boost/spirit/include/phoenix\_operator.hpp>

23 #include <boost/spirit/include/phoenix\_stl.hpp>

24

25 #include <boost/lexical\_cast.hpp>

26 #include <boost/fusion/include/adapt\_struct.hpp>

27 #include <boost/fusion/include/std\_pair.hpp>

28 #include <boost/config/warning\_disable.hpp>

29 #include <boost/spirit/include/qi.hpp>

30 #include <boost/spirit/include/qi\_numeric.hpp>

31 #include <boost/spirit/include/classic\_position\_iterator.hpp>

32 #include <boost/spirit/include/phoenix\_core.hpp>

33 #include <boost/spirit/include/phoenix\_function.hpp>

34 #include <boost/spirit/include/phoenix\_fusion.hpp>

35 #include <boost/spirit/include/phoenix\_object.hpp>

36 #include <boost/spirit/include/phoenix\_operator.hpp>

37 #include <boost/spirit/include/phoenix\_stl.hpp>

38 #include <boost/spirit/include/support\_multi\_pass.hpp>

39 #include <boost/tuple/tuple.hpp>

40 #include <boost/variant/apply\_visitor.hpp>

41 #include <boost/variant/recursive\_variant.hpp>

42

43 #include <stan/gm/ast.hpp>

44 #include <stan/gm/grammars/whitespace\_grammar.hpp>

45 #include <stan/gm/grammars/term\_grammar.hpp>

46 #include <stan/gm/grammars/expression\_grammar.hpp>

47 #include <stan/gm/grammars/expression07\_grammar.hpp>

48

49

50 namespace stan {

51

52  namespace gm {

53

54  struct validate\_expr\_type3 {

55  template <typename T1, typename T2>

56  struct result { typedef bool type; };

57

58  bool operator()(const expression& expr,

59  std::ostream& error\_msgs) const {

60  if (expr.expression\_type().is\_ill\_formed()) {

61  error\_msgs << "expression is ill formed" << std::endl;

62  return false;

63  }

64  return true;

65  }

66  };

67  boost::phoenix::function<validate\_expr\_type3> validate\_expr\_type3\_f;

68

69  // FIXME: cut and paste from term grammar, having trouble w. includes

70  struct set\_fun\_type3 {

71  template <typename T1, typename T2>

72  struct result { typedef fun type; };

73

74  fun operator()(fun& fun,

75  std::ostream& error\_msgs) const {

76  std::vector<expr\_type> arg\_types;

77  for (size\_t i = 0; i < fun.args\_.size(); ++i)

78  arg\_types.push\_back(fun.args\_[i].expression\_type());

79  fun.type\_ = function\_signatures::instance().get\_result\_type(fun.name\_,

80  arg\_types,

81  error\_msgs);

82  return fun;

83  }

84  };

85  boost::phoenix::function<set\_fun\_type3> set\_fun\_type3\_f;

86

87  struct addition\_expr3 {

88  template <typename T1, typename T2, typename T3>

89  struct result { typedef expression type; };

90

91  expression operator()(expression& expr1,

92  const expression& expr2,

93  std::ostream& error\_msgs) const {

94  if (expr1.expression\_type().is\_primitive()

95  && expr2.expression\_type().is\_primitive()) {

96  return expr1 += expr2;

97  }

98  std::vector<expression> args;

99  args.push\_back(expr1);

100  args.push\_back(expr2);

101  set\_fun\_type3 sft;

102  fun f("add",args);

103  sft(f,error\_msgs);

104  return expression(f);

105  return expr1 += expr2;

106  }

107  };

108  boost::phoenix::function<addition\_expr3> addition3\_f;

109

110

111  struct subtraction\_expr3 {

112  template <typename T1, typename T2, typename T3>

113  struct result { typedef expression type; };

114

115  expression operator()(expression& expr1,

116  const expression& expr2,

117  std::ostream& error\_msgs) const {

118  if (expr1.expression\_type().is\_primitive()

119  && expr2.expression\_type().is\_primitive()) {

120  return expr1 -= expr2;

121  }

122  std::vector<expression> args;

123  args.push\_back(expr1);

124  args.push\_back(expr2);

125  set\_fun\_type3 sft;

126  fun f("subtract",args);

127  sft(f,error\_msgs);

128  return expression(f);

129  }

130  };

131  boost::phoenix::function<subtraction\_expr3> subtraction3\_f;

132

133

134

135  template <typename Iterator>

136  expression07\_grammar<Iterator>::expression07\_grammar(variable\_map& var\_map,

137  std::stringstream& error\_msgs,

138  expression\_grammar<Iterator>& eg)

139  : expression07\_grammar::base\_type(expression07\_r),

140  var\_map\_(var\_map),

141  error\_msgs\_(error\_msgs),

142  term\_g(var\_map,error\_msgs,eg)

143  {

144  using boost::spirit::qi::\_1;

145  using boost::spirit::qi::char\_;

146  using boost::spirit::qi::double\_;

147  using boost::spirit::qi::eps;

148  using boost::spirit::qi::int\_;

149  using boost::spirit::qi::lexeme;

150  using boost::spirit::qi::lit;

151  using boost::spirit::qi::\_pass;

152  using boost::spirit::qi::\_val;

153  using boost::spirit::qi::labels::\_r1;

154

155  expression07\_r.name("expression, precedence 7, binary +, -");

156  expression07\_r

157  = term\_g(\_r1)

158  [\_val = \_1]

159  > \*( ( lit('+')

160  > term\_g(\_r1) // expression07\_r

161  [\_val = addition3\_f(\_val,\_1,boost::phoenix::ref(error\_msgs))] )

162  |

163  ( lit('-')

164  > term\_g(\_r1) // expression07\_r

165  [\_val = subtraction3\_f(\_val,\_1,boost::phoenix::ref(error\_msgs))] )

166  )

167  > eps[\_pass = validate\_expr\_type3\_f(\_val,boost::phoenix::ref(error\_msgs\_))]

168  ;

169

170

171  }

172  }

173 }

174

175 #endif

stan::gm::subtraction\_expr3

**Definition:** expression07\_grammar\_def.hpp:111

stan::gm::addition\_expr3

**Definition:** expression07\_grammar\_def.hpp:87

stan::gm::fun::name\_

std::string name\_

**Definition:** ast.hpp:351

stan::gm::set\_fun\_type3

**Definition:** expression07\_grammar\_def.hpp:70

stan::gm::fun::type\_

expr\_type type\_

**Definition:** ast.hpp:353

expression07\_grammar.hpp

stan::gm::function\_signatures::instance

static function\_signatures & instance()

**Definition:** ast\_def.cpp:143

expression\_grammar.hpp

stan::gm::expression\_grammar

**Definition:** expression07\_grammar.hpp:23

stan::gm::addition\_expr3::operator()

expression operator()(expression &expr1, const expression &expr2, std::ostream &error\_msgs) const

**Definition:** expression07\_grammar\_def.hpp:91

stan::gm::validate\_expr\_type3::result::type

bool type

**Definition:** expression07\_grammar\_def.hpp:56

stan::gm::validate\_expr\_type3::result

**Definition:** expression07\_grammar\_def.hpp:56

stan::gm::fun::args\_

std::vector< expression > args\_

**Definition:** ast.hpp:352

stan::gm::validate\_expr\_type3\_f

boost::phoenix::function< validate\_expr\_type3 > validate\_expr\_type3\_f

**Definition:** expression07\_grammar\_def.hpp:67

stan::gm::addition\_expr3::result

**Definition:** expression07\_grammar\_def.hpp:89

stan::gm::set\_fun\_type3::result

**Definition:** expression07\_grammar\_def.hpp:72

stan::gm::variable\_map

**Definition:** ast.hpp:439

stan::gm::expression07\_grammar::term\_g

term\_grammar< Iterator > term\_g

**Definition:** expression07\_grammar.hpp:39

stan::gm::expression07\_grammar::error\_msgs\_

std::stringstream & error\_msgs\_

**Definition:** expression07\_grammar.hpp:37

stan::gm::addition3\_f

boost::phoenix::function< addition\_expr3 > addition3\_f

**Definition:** expression07\_grammar\_def.hpp:108

stan::gm::expression

**Definition:** ast.hpp:216

stan::gm::expression07\_grammar::expression07\_grammar

expression07\_grammar(variable\_map &var\_map, std::stringstream &error\_msgs, expression\_grammar< Iterator > &eg)

**Definition:** expression07\_grammar\_def.hpp:136

stan::gm::set\_fun\_type3\_f

boost::phoenix::function< set\_fun\_type3 > set\_fun\_type3\_f

**Definition:** expression07\_grammar\_def.hpp:85

stan::gm::set\_fun\_type3::result::type

fun type

**Definition:** expression07\_grammar\_def.hpp:72

stan::gm::validate\_expr\_type3

**Definition:** expression07\_grammar\_def.hpp:54

term\_grammar.hpp

stan::gm::set\_fun\_type3::operator()

fun operator()(fun &fun, std::ostream &error\_msgs) const

**Definition:** expression07\_grammar\_def.hpp:74

ast.hpp

stan::gm::subtraction\_expr3::result::type

expression type

**Definition:** expression07\_grammar\_def.hpp:113

stan::gm::fun

**Definition:** ast.hpp:350

stan::gm::subtraction3\_f

boost::phoenix::function< subtraction\_expr3 > subtraction3\_f

**Definition:** expression07\_grammar\_def.hpp:131

stan::gm::expr\_type::is\_ill\_formed

bool is\_ill\_formed() const

**Definition:** ast\_def.cpp:105

whitespace\_grammar.hpp

stan::gm::subtraction\_expr3::operator()

expression operator()(expression &expr1, const expression &expr2, std::ostream &error\_msgs) const

**Definition:** expression07\_grammar\_def.hpp:115

stan::gm::addition\_expr3::result::type

expression type

**Definition:** expression07\_grammar\_def.hpp:89

stan::gm::subtraction\_expr3::result

**Definition:** expression07\_grammar\_def.hpp:113

stan::gm::expression07\_grammar::expression07\_r

boost::spirit::qi::rule< Iterator, expression(var\_origin), whitespace\_grammar< Iterator > > expression07\_r

**Definition:** expression07\_grammar.hpp:45

stan::gm::function\_signatures::get\_result\_type

expr\_type get\_result\_type(const std::string &name, const std::vector< expr\_type > &args, std::ostream &error\_msgs)

**Definition:** ast\_def.cpp:329

stan::gm::validate\_expr\_type3::operator()

bool operator()(const expression &expr, std::ostream &error\_msgs) const

**Definition:** expression07\_grammar\_def.hpp:58

stan::gm::expression07\_grammar

**Definition:** expression07\_grammar.hpp:26

stan::gm::expression::expression\_type

expr\_type expression\_type() const

**Definition:** ast\_def.cpp:570

stan::gm::expr\_type::is\_primitive

bool is\_primitive() const

**Definition:** ast\_def.cpp:93


---

[ Stan Home Page ]

*© 2011–2014,
Stan Development Team.*
